# Integrated validation of a cross-cultural emotional competence scale using Confirmatory Factor Analysis, Rasch Modeling and Exploratory Graph Analysis

**DOI:** 10.1016/j.mex.2025.103627

**Published:** 2025-09-15

**Authors:** Angélica Garzón-Umerenkova, Marisleidy Alba, Boris Cendales, Juan Carlos Espinosa, Janitza Ariza

**Affiliations:** a Facultad de Psicología, Fundación Universitaria Konrad Lorenz, Bogotá D.C., Colombia; b Escuela de Negocios, Fundación Universitaria Konrad Lorenz, Bogotá D.C., Colombia; c Facultad de Ciencias Económicas y Administrativas, Universidad El, Bosque. D.C., Colombia; d Escuela de Administración, Universidad del Rosario, Bogotá D.C., Colombia

**Keywords:** Psychometric methods, Cross-Cultural adaptation, Confirmatory Factor Analysis, Rasch Modeling, Exploratory Graph Analysis, Likert scale functioning, Emotional Competence, Self-awareness, Self-regulation, Latin America, College Students

## Abstract

This study validates the Emotional Competence Questionnaire (ECQ) in five Latin American countries (Peru, Mexico, Ecuador, Paraguay, and Colombia) using a multi-method psychometric protocol. To ensure cultural sensitivity, structural validity, and methodological rigor, three complementary approaches were integrated: Confirmatory Factor Analysis, Rasch modeling, and Exploratory Graph Analysis. This protocol provides a reproducible framework for evaluating psychological instruments across cultures, balancing theoretical alignment with empirical evidence. The results highlight both the strengths and areas for refinement of the ECQ, offering practical guidance for future cross-cultural adaptations.

## Highlights

• Confirmatory Factor Analysis supported the theoretical five-factor structure of the ECQ with acceptable fit indices.
• Rasch analysis evaluated item functioning and confirmed the suitability of a 5-point Likert scale.
• Exploratory Graph Analysis identified four emergent dimensions, suggesting overlap between self-awareness and self-regulation, and flagged one unstable item.

## Graphical abstract

**Figure:**
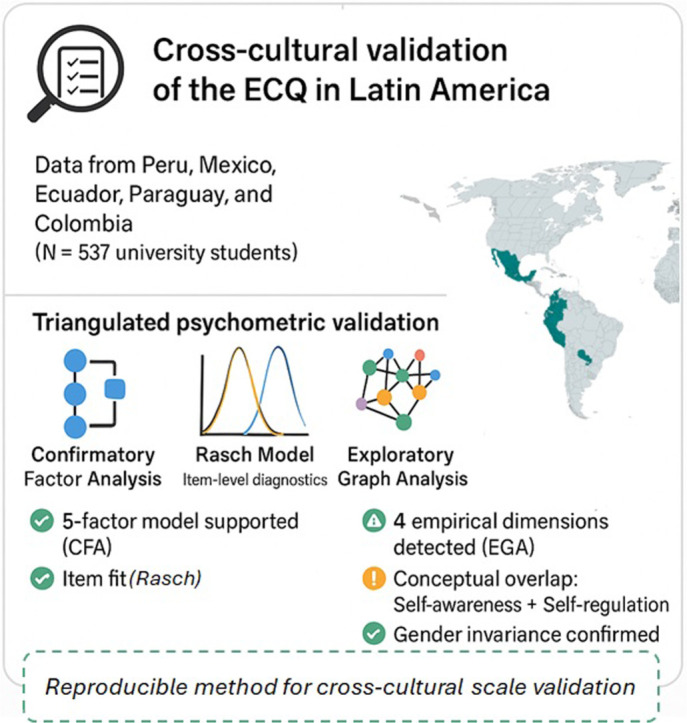

## Specifications table

**Table:** 

**Subject area**	Psychology
**More specific subject area**	Psychometrics
**Name of your method**	Integrated validation of a cross-cultural emotional competence scale using Confirmatory Factor Analysis, Rasch Modeling and Exploratory Graph Analysis
**Name and reference of original method**	NA
**Resource availability**	*A. Garzón-Umerenkova, É.-A. Malpica-Chavarria, J. Gil-Flores, Caracterización psicométrica de la prueba de procrastinación PASS en alumnos de educación secundaria colombianos, Estud. Sobre Educ. 48 (2025) 7–33. https://doi.org/10.15581/004.48.001* *H. Golino, A.P. Christensen, L.E. Garrido, Invited commentary: Exploratory graph analysis in context, Rev. Psicol. Teor. Prát. 24 (3) (2022). https://doi.org/10.5935/1980-6906/ePTPIC15531.en*

## Background

Emotional competencies (EC) are observable abilities related to the perception, regulation, and appropriate expression of emotions. They are fundamental for psychological well-being, academic adjustment, and professional success. In recent years, EC have gained increasing relevance in the field of entrepreneurship education, where self-motivation and emotional regulation are recognized as key drivers in the transition from entrepreneurial intention to action [1]. Entrepreneurship involves navigating uncertainty, making high-stakes decisions under pressure, and managing complex social relationships in dynamic environments [2,3].

A growing body of literature has shown that the development of EC enhances critical entrepreneurial traits such as self-efficacy, perseverance, empathy, adaptability, and initiative. These skills play a crucial role in identifying and exploiting entrepreneurial opportunities. As such, the rigorous assessment of EC is essential for designing effective educational interventions that foster comprehensive entrepreneurial profiles—particularly in Latin America, where culturally contextualized and locally validated instruments are still scarce.

Despite the growing interest in emotional competencies, most instruments used to assess them have been developed in European [2] or North American contexts [4], often without adequate psychometric validation for Latin American populations. Frequently used instruments include the Profile of Emotional Competence (PEC) [4], the Trait Meta-Mood Scale (TMMS) [5], and Bar-On’s Emotional Intelligence model [6], widely applied in educational and organizational settings [2]. However, the Emotional Competence Questionnaire (ECQ) developed by [2], specifically designed to assess competencies relevant to entrepreneurial processes, has not yet been formally validated for use in Latin America. This limits both international comparability and the ability to evaluate EC in culturally meaningful ways across the region.

To address this gap, the present study proposes a novel and replicable methodological approach for validating the ECQ among university students in five Latin American countries (Peru, Mexico, Ecuador, Paraguay, and Colombia). It consists of 21 items designed to evaluate observable emotional capacities that are essential for individual and entrepreneurial development.

Our validation strategy integrates three complementary analytical approaches, enhancing the robustness, depth, and applicability of the psychometric assessment:

1. Confirmatory Factor Analysis (CFA), grounded in Classical Test Theory, to evaluate the theoretical structure and overall model fit.
2. The Polytomous Rasch Model, from Item Response Theory, which examines dimensionality, category functioning, item difficulty, and Differential Item Functioning (DIF), ensuring fairness and accuracy in diverse populations.
3. Exploratory Graph Analysis (EGA), a network-based exploratory method that estimates the empirical dimensional structure without prior assumptions, identifies natural clusters of items, and assesses their stability via bootstrap resampling [7].

Unlike traditional approaches that rely on a single validation method, this integrated design provides a more holistic and empirically triangulated view of the instrument’s structure, offering advantages for cross-cultural adaptations. The combination of confirmatory, probabilistic, and network-based methods enables the detection of item redundancy, conceptual overlap, and multidimensionality issues, while ensuring the functional equivalence of items across groups.

This methodology provides a transparent and reproducible protocol for adapting psychometric instruments in multicultural contexts. It is especially relevant for studies that combine structural validation with applied research in educational and entrepreneurial settings, contributing to the development of culturally sensitive tools that support evidence-based educational innovation in Latin America.

## Method details

This protocol outlines a comprehensive and replicable methodology for adapting and validating the Emotional Competence Questionnaire (ECQ) in Latin American university settings. The approach integrates multiple psychometric techniques to ensure structural validity, measurement invariance, and dimensional stability, providing a robust framework for researchers and educators in cross-cultural contexts.

### Instrument adaptation and cultural adjustment

To assess emotional competence, we used the scale developed by [2], which consists of 21 items grouped into five dimensions: *Self-awareness (3 items), Self-regulation (4 items), Motivation (4 items), Empathy (5 items),* and *Social skills (5 items)*.

The psychometric validation of the instrument was carried out by [2] on a sample of Spanish university students, within the context of courses on business creation and entrepreneurship. Confirmatory Factor Analysis (CFA) supported the five-factor structure corresponding to the proposed emotional competencies, with an adequate goodness of fit (example: χ²/df = 3.78, GFI = 0.986, CFI = 0.903, RMSEA = 0.061).

The results demonstrated both convergent and discriminant validity among the factors, as well as the instrument's suitability for individual assessment and for analyzing changes in emotional competencies following educational interventions in entrepreneurship. Moreover, multi-group analyses indicated that the structure and functioning of the instrument remained stable in both pre- and post-intervention measurements.

The adaptation process carried out in this research followed the TRAPD methodology (Translation, Review, Adjudication, Pretesting, and Documentation), as recommended for rigorous cross-cultural validation [8]. Since the original ECQ was already in Spanish (Spain), translation was not necessary, but cultural and linguistic adjustments were required. A panel of experts in psychology and psychometrics reviewed the items to resolve discrepancies and ensure cultural equivalence. Through the adjudication process, a consensus version was reached. The original 7-point Likert scale was modified to a 5-point format (1 = Strongly disagree; 5 = Strongly agree) to improve clarity and suitability for the target population. The adapted version was pilot tested with university students, and minor revisions were made based on their feedback. All steps were documented to ensure transparency.

### Participants and data collection

The study involved 537 valid participants from five Latin American countries (Peru, Mexico, Ecuador, Paraguay, and Colombia), after removing 55 invalid responses from an initial total of 592. Participants were business school students from ACBSP-accredited institutions. The sample included 243 men (45.25%) and 294 women (54.75%), with age ranges as follows: 18–24 years (n = 462), 25–39 years (n = 74), and 40–69 years (n = 1). Prior experience in entrepreneurship was reported by 333 participants.

Data collection was conducted online through institutional authorization. Participation was voluntary and based on informed consent. Ethical approval was granted by the Bioethics Committee of Fundación Universitaria Konrad Lorenz (August 15, 2024).

### Data preprocessing

Before analysis, the data were screened for missing values and response quality. This preprocessing step was essential to ensure the accuracy of subsequent analyses. The analyses were conducted using Jamovi software, which supported exploratory factor analysis, reliability estimation, and nonparametric comparisons.

### Confirmatory Factor Analysis (CFA)

A CFA was performed to validate the theoretical five-factor structure of the ECQ, using the Diagonally Weighted Least Squares (DWLS) estimator, appropriate for ordinal data, to confirm the factorial structure. The analysis was conducted using Jamovi. The results confirmed five distinct dimensions: Self-awareness, Self-regulation, Motivation, Empathy, and Social Skills.

### Rasch analysis

Following CFA confirmation results, a multidimensional Rasch analysis was conducted to assess the five-dimensional structure. The results indicated the existence of one latent attribute, not five. So, the unidimensional analysis was performed with the 21 items, and the Partial Credit Model (PCM) for polytomous Likert-type items was applied. Key procedures included unidimensionality, local independence, item fit using Infit and Outfit statistics, Weighted Likelihood Estimation (WLE), and Expected a Posteriori (EAP) estimation, reliability, detection of Differential Item Functioning (DIF) by gender using Lord’s method with logistic regression and adjusted chi-square tests, and response category functioning analysis. R packages used: TAM [9], eRm [10], psych [11], tidyverse [12], and lordif [13].

### Network-based dimensional analysis: EGA and bootEGA

To empirically estimate the dimensional structure of the ECQ, Exploratory Graph Analysis (EGA) was conducted using psychometric network models (GLASSO or TMFG). Stability was evaluated using bootstrap EGA (bootEGA), which estimated the proportion of replications for each dimension count and the consistency of item assignments. Unique Variable Analysis (UVA) was also applied to detect redundant items. R packages used: EGAnet [14], psych [15], ggplot2 [16], and readr [17].

**Fig. 1:**
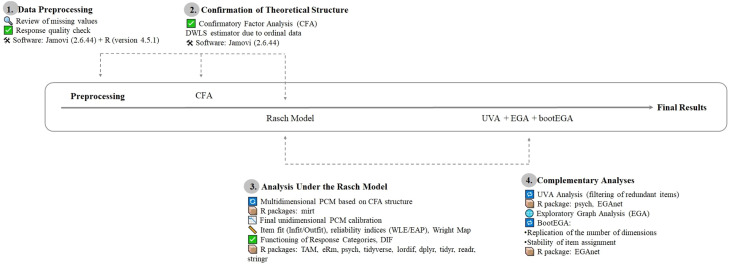
Methodological process.

The complete methodological pipeline for the psychometric validation of the Emotional Competence Questionnaire (ECQ) in Latin American university contexts. This is a visual guide organized into five numbered blocks that summarize the methodological steps of the entire validation process, from preprocessing to final validation, integrating CFA, Rasch analysis, and network-based methods (Fig. 1).

This multi-stage approach ensures replicability, technical transparency, and robust cross-cultural validation of the ECQ in Latin American university contexts. Likewise, the methodological strategy presented is replicable for adapting and psychometrically validating other instruments that assess psychological, educational, or behavioral variables, especially in studies requiring rigorous analyses in multicultural contexts.

## Method validation

As a result of the methodological application (Fig. 1), model fit was assessed using several indices: the Comparative Fit Index (CFI = 0.980), the Tucker-Lewis Index (TLI = 0.977), the Normed Fit Index (NFI = 0.976), and the Goodness of Fit Index (GFI = 0.999), all of which exceeded the recommended threshold of 0.90. The RMSEA was 0.093, with a 90% confidence interval between 0.087 and 0.099, and an SRMR of 0.026, indicating acceptable fit. The sample size was adequate according to Hoelter’s critical N (N = 295.79 at α = .05). Additionally, all Average Variance Extracted (AVE) values exceeded the 0.50 threshold, confirming adequate convergent validity. Internal consistency was also assessed using composite reliability (rho_A and rho_C), with all values above 0.70, confirming the internal reliability of the scales [18].

The results supported the five-factor structure, showing acceptable fit indices: χ²(179) = 1011.823, *p* < .001, CFI = 0.980, TLI = 0.977, RMSEA = 0.093, SRMR = 0.026. All factor loadings were statistically significant (*p* < .001) and above 0.96 for most items.

The five-correlated factor model showed that all ECQ dimensions were significantly related to each other (Table 1). The correlations ranged from moderate to high, with values from .63 (SR–SS) to .96 (SA–SR). Very high correlations were observed between Self-Management (SA) and Social Regulation (SR) (r = .964), as well as between Empathy (E) and Emotional Awareness (EC) (r = .944). On the other hand, more moderate correlations were found between Social Regulation (SR) and Social Support (SS) (r = .632). These results indicate that the ECQ subscales are positively associated, reflecting the theoretical coherence of the construct [2].

**Table 1:** Latent factor correlation matrix (with strength and direction).

	E	EC	M	SA	SR	SS
E		0.944	0.772	0.696	0.712	0.751
EC			0.927	0.898	0.883	0.915
M				0.801	0.818	0.713
SA					0.964	0.686
SR						0.632

Note. All correlations are positive. Values ≥ .70 are interpreted as high; between .40 and .69 as moderate; ≤ .39 as low.

### Rasch model fit analysis

A multidimensional Rasch analysis was performed according to the five factors obtained with the CFA. To this Rasch analysis, we follow the methodological recommendations of [[19], [20], [21]] using the partial credit model and performed a scale alignment process [22,23] to ensure that scores for each subscale are comparable. The analysis was performed through MIRT [24], which was used for the estimation of the multidimensional generalized partial credit model (PCM) constrained to Rasch parameterization by fixing slopes to 1. The package allowed for the flexible specification of multidimensional structures, estimation through the Metropolis–Hastings Robbins–Monro (MHRM) algorithm, and the computation of expected a posteriori (EAP) score with quasi–Monte Carlo (QMC) integration. The five-dimensional Rasch model (Self-awareness, Self-regulation, Motivation, Empathy, and Social Skills) yielded very high latent correlations among all factors. The Phi coefficients ranged from .85 to .97, indicating a substantial degree of overlap across subscales. These results suggest that the five factors do not operate as clearly distinct constructs but rather share a large proportion of their variance. From a measurement perspective, this provides strong evidence for the presence of a single underlying latent attribute that accounts for item responses across the instrument.

Thus, although the confirmatory factor analysis supported a five-factor correlated structure, the multidimensional Rasch analysis revealed that the dimensions are so highly interrelated that the scale can be considered essentially unidimensional. Consequently, the 21 items altogether were analyzed. Unidimensionality, local independence, item fit, and reliability were evaluated. The Partial Credit Model (PCM) was estimated with the TAM package [9], from which we obtained item fit statistics (Infit/Outfit; tam.fit), person scores (WLE/EAP), reliability indices, and category thresholds. Person–item maps were produced with WrightMap, and data wrangling and tabulation were performed with dplyr, tidyr, readr, and stringr [25].

Principal components parallel analysis confirmed the unidimensionality. The principal component analysis of the residuals (PCA) revealed that the first three contrasts had eigenvalues above 2 (4.12, 2.84, and 2.15), which could indicate the presence of substructures within the scale. However, parallel analysis (p95), based on parametric simulations, showed that none of these contrasts exceeded the 95th percentile expected under the fitted Rasch model. Consequently, the residual groupings are likely attributable to chance, supporting the interpretation that the 21 items share a single latent attribute and that, for analysis purposes, the scale can be treated as essentially unidimensional.

Local independence was assessed through residual correlation analysis (Q3 index). No high residual correlations were detected in most item pairs, suggesting that the assumption of local independence is generally met. In the shorter subscales (three items) and in some specific cases, high simple correlations between items were observed, indicating possible redundancies. Due to these elevated correlations in certain item pairs, a redundancy analysis was additionally conducted using Unique Variable Analysis (UVA), which helps identify conditionally redundant item pairs within the correlation network. UVA results did not detect significant “unique” redundancies under the standard criterion (cut-off = 0.25), indicating that the observed relationships between items can be explained by the overall structure of the instrument rather than by direct duplication between pairs. The UVA results indicated no redundancies between items, thus supporting the assumption of local independence.

Furthermore, the fit results of the polytomous Rasch model showed that all items fell within the acceptable range for individual item fit. With most items falling within the acceptable fit range (0.7–1.3). These values are expressed in Mean Square (MNSQ) [26,27]. This indicates that most items were consistent with the expectations of the Rasch model, although a small subset showed misfit and may require further review. The slowly overfitting items (<0.7), which are predictable, are *SS5, E1,* and *SS2*. The slowly underfit items (>1.3), which are ambiguous, are *SR3, SR4*, and *M3*. The adjustment difficulties mainly belong to the SR and SS factors.

However, these results support the psychometric quality of the instrument at the item level, indicating that each item contributes appropriately to measuring its respective dimension. (Table 2)

**Table 2:** Infit and outfit estimations for each item by subscale.

	Measure	S.E.Measure	Infit	Outfit
EC-SA1	-4.7	0.09	1.02	1.05
EC-SA2	-4.8	0.09	1.01	0.99
EC-SA3	-4.3	0.08	1.07	1.12
EC-SR1	-4.8	0.09	1.20	1.25
EC-SR2	-4.9	0.09	1.18	1.22
EC-SR3	-4.6	0.08	1.31	1.35
EC-SR4	-4.6	0.09	1.34	1.38
EC-M1	-4.3	0.08	0.95	0.92
EC-M2	-4.8	0.09	1.08	1.10
EC-M3	-4.2	0.08	1.26	1.34
EC-M4	-4.5	0.08	1.12	1.15
EC-E1	-4.6	0.09	0.89	0.85
EC-E2	-4.6	0.09	1.05	1.08
EC-E3	-4.2	0.08	1.15	1.18
EC-E4	-4.7	0.09	1.09	1.11
EC-E5	-4.5	0.09	1.04	1.07
EC-SS1	-3.8	0.08	0.97	0.94
EC-SS2	-4.4	0.08	0.93	0.91
EC-SS3	-4.1	0.08	1.10	1.14
EC-SS4	-4.0	0.08	1.22	1.28
EC-SS5	-4.0	0.08	0.72	0.70

The person reliability indices were acceptable, with Weighted Likelihood Estimation (WLE = 0.76) and Expected a Posteriori estimation (EAP = 0.78), corresponding to a person separation index of approximately 1.8–1.9, indicating that the instrument can distinguish about two ability levels among respondents. Item reliability was excellent (≈ 0.90), with an item separation index of approximately 3.0, indicating that the sample size and variability were sufficient to confirm stable calibrations, allowing items to be reliably ordered along the latent continuum. Person and item reliability are adequate in estimating the latent levels of emotional competence among participants [28,29].

### Additional Rasch analyses: Parameter b, DIF, and Functioning of Response Categories

Once the test fit to Rasch model expectations was established, additional analyses were conducted to examine construct validity, differential item functioning (DIF) by gender, and the functioning of the Likert scale response categories.

**Fig. 2:**
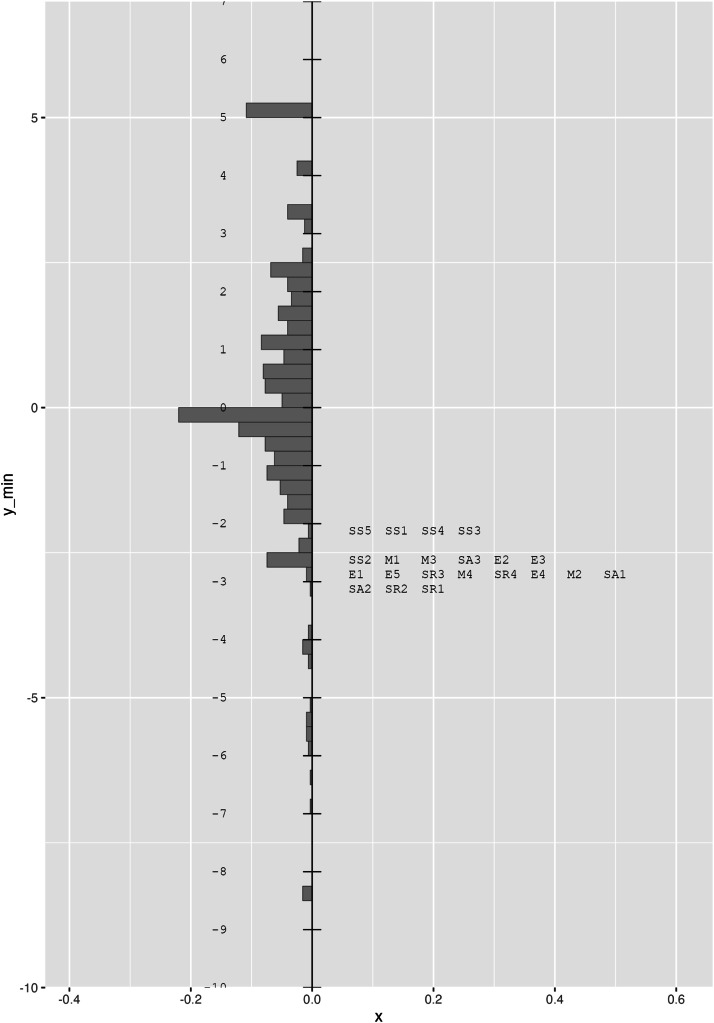
Wright map.

Construct validity evidence was assessed through the item difficulty hierarchy, estimated and visually represented in person-item maps (Wright maps). In these graphs, items are placed on the right axis and participants on the left, allowing for an assessment of the alignment between item difficulty and the respondents’ latent ability. Ideally, items should be distributed across the ability continuum—from the easiest at the bottom to the most difficult at the top [25]. The Wright map showed an adequate distribution of item difficulty and participant ability (See Fig. 2). In this case, most of the items are located at the bottom level, which suggests that the test could be improved by incorporating more difficult new items that cover a higher level of emotional competence attribute.

A differential item functioning (DIF) analysis by gender was also conducted using the ordinal logistic regression method proposed by Lord and using the lordif package [13], with the aim of detecting potential bias in item responses on the scale. The results indicated no evidence of uniform or non-uniform DIF in any of the 21 items (all Benjamini–Hochberg adjusted p-values > .95). These findings support the invariance of the instrument across male and female respondents, confirming that the items function equivalently across groups.

Finally, to verify the adequacy of the Likert-type response format used (categories: 1 = strongly disagree, 2 = disagree, 3 = neither agree nor disagree, 4 = agree, 5 = strongly agree), each item has a delta value (general difficulty) and four thresholds (τ1–τ4). The results, as shown in Table 3, indicate that response categories 2, 3, and 4 received the most responses, while the extreme categories (1 and 5) were used by the participants to a lesser extent. These values indicate that a very low level of emotional competence is required for a respondent to choose the first category, which is common in instruments administered to university populations, where there is often a tendency toward higher responses [28].

**Table 3:** Functioning of the five response categories.

Item	Delta	tau1	tau2	tau3	tau4	Monotonic
**EC.E1**	-2.817	-6.019	-4.259	-2.633	1.643	True
**EC.E2**	-2.731	-5.45	-4.532	-2.672	1.731	True
**EC.E3**	-2.733	-6.431	-4.644	-1.881	2.025	True
**EC.E4**	-2.896	-5.819	-4.702	-2.814	1.75	True
**EC.E5**	-2.836	-5.786	-4.88	-2.444	1.767	True
**EC.M1**	-2.573	-4.983	-4.766	-2.167	1.624	True
**EC.M2**	-2.936	-4.577	-5.217	-3.097	1.148	False
**EC.M3**	-2.574	-5.308	-4.545	-2.095	1.651	True
**EC.M4**	-2.879	-5.06	-5.403	-2.374	1.322	False
**EC.SA1**	-2.967	-5.794	-4.005	-3.301	1.234	True
**EC.SA2**	-3.035	-5.162	-4.879	-3.428	1.328	True
**EC.SA3**	-2.593	-5.16	-4.31	-2.464	1.561	True
**EC.SR1**	-3.146	-6.226	-4.263	-2.817	722	True
**EC.SR2**	-3.112	-5.37	-4.42	-3.421	762	True
**EC.SR3**	-2.873	-5.559	-4.236	-2.759	1.061	True
**EC.SR4**	-2.896	-5.249	-4.357	-3.016	1.04	True
**EC.SS1**	-2.131	-6.146	-3.726	-1.217	2.564	True
**EC.SS2**	-2.547	-5.883	-4.167	-2.206	2.068	True
**EC.SS3**	-2.248	-5.829	-3.922	-1.689	2.448	True
**EC.SS4**	-2.134	-5.452	-4.018	-1.244	2.178	True
**EC.SS5**	-2.044	-4.898	-4.034	-1.437	2.191	True

Approximately 80% of the items have arranged thresholds (τ1 < τ2 < τ3 < τ4), while some items have unarranged thresholds mainly between 2-3 and 4-5 categories. In most items, the thresholds moved in a logical form (from negative to positive values), which indicates that the scale measured a continuous attribute (emotional competence). However, in unarranged threshold items (M2, M4), the middle categories overlap and do not give measurement information.

In conclusion, the five-point Likert scale could be useful, but it could be improved. For instance, future research may consider collapsing adjacent categories or improving the wording of anchors to encourage the use of extreme categories.

### Complementary statistical analyses: EGA and bootEGA

Finally, as a complementary analysis, Exploratory Graph Analysis (EGA) and its bootstrap version (bootEGA) were applied to explore the “natural” dimensional structure of the scale, considering the complete set of items. EGA identifies item communities that form latent dimensions based on the correlation network, without assuming a theoretical structure. This approach improves the validity of the estimated parameters and the interpretation of structural relationships, avoiding overlap and multicollinearity that may arise from using highly correlated theoretical subscales.

**Fig. 3:**
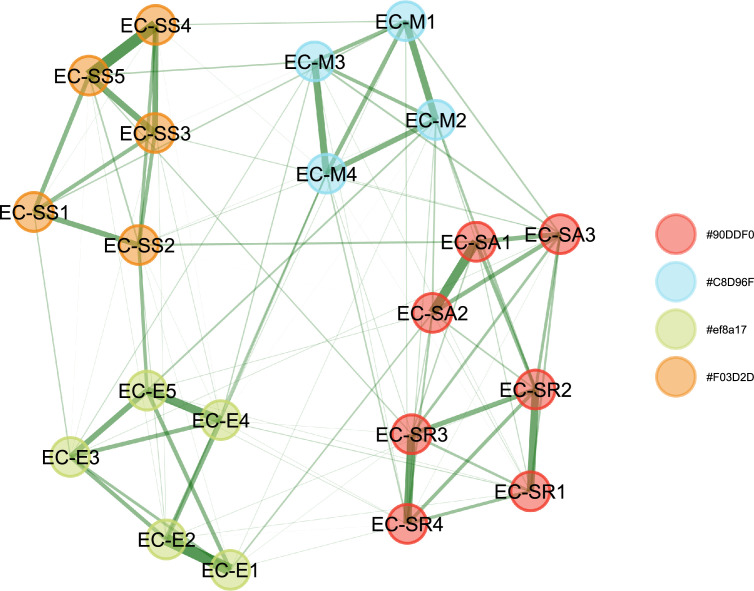
Exploratory Graph Analysis.

In the total sample, EGA identified four main communities, which contrasts with the five theoretical dimensions of the instrument. As shown in Fig. 3, the items related to self-awareness and self-regulation merged into a single community. This means that, according to EGA, the content of these items is similar, which is why they are grouped together. In contrast, the items for Motivation, Social Skills, and Empathy maintained the item communities from the original test structure.

**Fig. 4:**
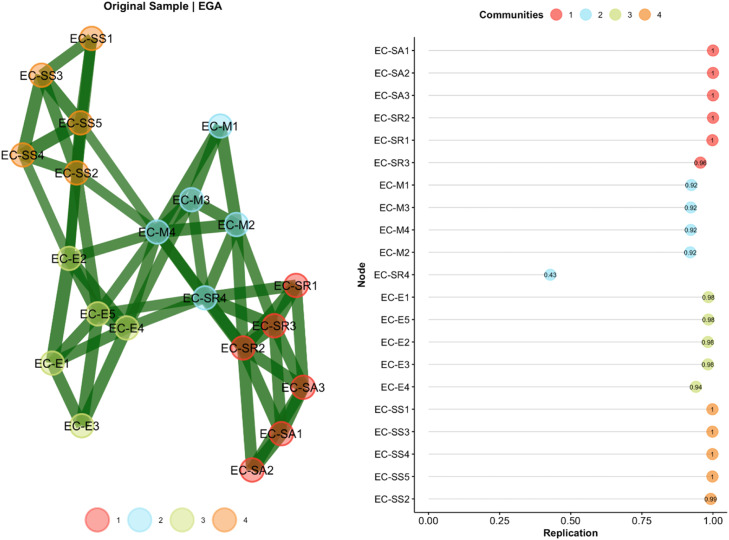
Exploratory Graph Analysis (bootstrap).

The bootEGA analysis (see Fig. 4) confirmed the high stability of this solution, showing that in more than 90% of the bootstrap samples, the four-dimensional structure was replicated, while in a smaller proportion (around 10%), three dimensions were identified. The 95% confidence interval for the number of dimensions was [3.43, 4.57], supporting the robustness of the empirical structure found.

Additionally, the bootEGA results revealed that one item showed a less stable assignment to the detected communities—item SR4 (“I feel comfortable and open to new ideas, approaches, and information”)—acting as a “border item” between dimensions. This finding suggests the presence of conceptual overlaps or potential areas for improving the scale’s internal differentiation.

Beyond its practical applicability, the protocol employed in this study offers a replicable and transparent methodological approach for psychometric validation in multicultural contexts. The sequential integration of three complementary approaches—Confirmatory Factor Analysis (CFA), the Polytomous Rasch Model, and Exploratory Graph Analysis (EGA/bootEGA)—allows not only for the assessment of theoretical structural validity but also for the empirical exploration of latent dimensionality and item functioning. This methodological triangulation provides a robust pathway that can be adapted by other researchers to validate instruments in new cultural or linguistic populations, ensuring both theoretical comparability and empirical optimization of the model. Moreover, the use of open-source software (Jamovi, R, and its specialized packages) facilitates the technical reproducibility of each stage, promoting good open science practices in psychometrics.

Discrepancies between classical factor analysis and Rasch dimensional results are frequently reported in the literature due to the differing statistical assumptions underlying each approach. These apparent discrepancies are not contradictory, but rather complementary. In this case, the CFA results indicate that items cluster according to the theorized subscales, while the Rasch analysis shows that these subscales are so strongly interrelated that they essentially reflect a single latent continuum [30]. In other words, the instrument conceptually differentiates five facets of emotional competence, but empirically, the responses are largely driven by one common underlying attribute.

Regarding the potential uses of the Emotional Competence scale for measuring and assessing emotional competencies, there are two recommended applications. On one hand, if the goal is to maximize psychometric precision and minimize measurement error, it is advisable to use the empirical structure detected through psychometric network analysis (EGA/bootEGA), which groups items into four independent dimensions and reduces redundancy. This provides more realistic standard error estimates and scores that are less inflated by overlap between dimensions. On the other hand, if theoretical interpretation and comparability with previous studies are a priority, the original five-subscale structure may be retained, while acknowledging the potential redundancy between the Self-Regulation and Self-Awareness dimensions. From a psychometric standpoint, both approaches to using the scale (with five or four dimensions) are supported.

## Limitations

While this study provides a robust and replicable methodology for validating the Emotional Competence Questionnaire (ECQ) in Latin American university settings, several limitations should be acknowledged. First, although the sample includes five culturally diverse countries, all participants were enrolled in business programs, which may limit the generalizability of the findings to other academic disciplines or age groups. Second, the cross-sectional nature of the data does not allow for the assessment of test–retest reliability or longitudinal changes in emotional competence. Further studies could explore these issues using longitudinal or intervention designs, as well as applications in broader educational, clinical, or organizational settings.

## Related research article

None.

## Ethics statements

To ensure ethical compliance, this study followed institutional protocols governing educational research. Prior to participation, all subjects gave their consent to be included in the study and for their responses to be used for research purposes. This study was conducted in accordance with the principles of the Declaration of Helsinki. The Ethics Committee of the Konrad Lorenz University Foundation granted approval on August 15, 2024.

It is confirmed that informed consent was obtained from all participants and/or their legal guardians. Participation in the study is completely voluntary, and the research instrument used will not cause discomfort, physical, or mental harm to the participants. The research will be conducted fairly and equitably, ensuring that both men and women are included in the sample without gender discrimination. Additionally, the information obtained will be kept confidential and anonymous, in compliance with Decree 1377 of 2013, Law 1581 of 2012, and Andean Decision 351 of 1993, as well as the institutional policy on the handling of personal data.

## Declaration of competing interest

The authors declare that they have no known competing financial interests or personal relationships that could have appeared to influence the work reported in this paper.

## Data Availability

Data will be made available on request.
